# Prevalence and genetic diversity of *porcine reproduction and respiration syndrome viruses* in Sichuan Province from 2023–2024

**DOI:** 10.3389/fvets.2025.1618962

**Published:** 2025-08-06

**Authors:** MingXiang Li, Min Wang, Hao Li, KeLei Zhou, ZhiQiang Hu, WenXing Bai, GuiYing Hao, GuangWen Yan

**Affiliations:** ^1^College of Animal Science, Xichang University, Xichang, China; ^2^Key Laboratory of Animal Epidemic Disease Detection and Prevention in Panxi District, Sichuan, China; ^3^Academy of Agricultural Sciences of Liangshan Yi Autonomous Prefecture, Xichang, China; ^4^Agricultural and Rural Bureau of Liangshan Yi Autonomous Prefecture, Xichang, China

**Keywords:** *porcine reproductive and respiratory syndrome virus* (PRRSV), phylogenetic analysis, lineage 1.8, PRRSV-1, ORF5

## Abstract

**Introduction:**

Porcine reproductive and respiratory syndrome (PRRS), caused by *porcine reproductive and respiratory syndrome virus* (PRRSV), occurs frequently in China, and severely hinders the healthy development of the pig farming industry.

**Methods:**

To determine the genetic diversity and epidemiological characteristics of PRRSV strains in Sichuan Province, we collected 499 clinical samples suspected of PRRSV infection from 101 pig farms in 19 cities from 2023 to 2024.

**Results and Discussion:**

Among the 499 samples, 162 were positive for PRRSV, with a total prevalence of 32.46% according to RT-qPCR. Among the 101 pig farms, 55 were positive farm, resulting in a rate of 54.46%. Further analysis of the complete ORF5 gene sequences of 56 PRRSV strains revealed that they could be classified into six lineages: PRRSV-1, lineage 8 (HP-PRRSV), lineage 5 (Classical PRRSV), lineage 1.8 (NADC30-like strain), lineage 1.5 (NADC34-like strain), and lineage 3.5 (QYYZ-like strain). Notably, both the lineage 8 and PRRSV-1 strain were detected in the same sample, indicating the presence of mixed infection. This study revealed the coexistence of multiple lineages of PRRSV in Sichuan Province, with the lineage 1.8 emerging as the predominant epidemic lineage. The concurrent prevalence of multiple lineages underscores the importance of selecting matching vaccines on the basis of locally prevalent strains and the need for continuous epidemiological monitoring of PRRSV.

## 1 Introduction

PRRSV is one of the most economically devastating pathogens in swine production worldwide (1, 2). The disease is highly contagious, and is characterized by reproductive failure in sows (including abortions, stillbirths, and weak neonates) and severe respiratory distress in growing pigs (manifesting as dyspnea, fever, and high mortality) (3, 4). Despite extensive control efforts, PRRSV remains difficult to manage due to its rapid genetic evolution, sophisticated immune evasion mechanisms, and the limited cross-protection offered by current vaccines, including modified live virus (MLV) strains (5).

PRRSV is an enveloped, positive-sense RNA virus belonging to the order *Nidovirales*, family *Arteriviridae*, with a genome of approximately 15 kb (5–8). The viral genome contains at least 10 open reading frames (ORFs), among which ORF5 encodes the major envelope glycoprotein GP5, a highly variable antigenic target that serves as a key marker for PRRSV genotyping and molecular epidemiology (9, 10). Consequently, ORF5 sequence analysis has become a cornerstone for tracking PRRSV genetic diversity and classifying circulating strains (11). PRRSV is divided into two distinct genotypes: PRRSV-1 (European, Type I) and PRRSV-2 (North American, Type II), each genotype containing multiple genetic lineages (12, 13). The emergence of highly pathogenic PRRSV (HP-PRRSV, lineage 8) in 2006, marked by a 30-amino-acid deletion in non-structural proteins 2 (NSP2), triggered a nationwide outbreak with abortion rates of 30–50% in sows and mortality rates of 20–100% in piglets (14, 15). Currently, PRRSV-2 is phylogenetically classified into five major lineages in China: lineage 1.8 (NADC30-like), lineage 1.5 (NADC34-like), lineage 3 (QYYZ and GM2), lineage 5 (VR2332 and BJ-4), and lineage 8 (CH-1a and JXA1) (16, 17). The epidemiology of PRRSV in China has undergone significant shifts over time. Recent surveillance data indicate that PRRSV-2 still predominates, with lineage 1.5 and 1.8 remaining the most prevalent (18).

In contrast, PRRSV-1 strains, although less common, have shown increasing genetic diversification in China. These strains are classified into four lineages: BJEU06-1-like, Amervac-like, HKEU16-like, and NMEU09-1-like, with BJEU06-1-like emerging as the dominant lineage (58.1% by ORF5 analysis; 60% by whole-genome analysis) (19–21). Notably, three new PRRSV-1 lineages have been identified since 2018, suggesting accelerated viral evolution in recent years. While PRRSV-1 is traditionally considered less virulent, recent evidence indicates increasing pathogenicity, necessitating close monitoring (22).

Given the enduring threat posed by PRRSV to swine production, this study investigated the current molecular epidemiology of PRRSV in Sichuan Province from 2023 to 2024. We screened 499 clinical samples by RT-qPCR and sequenced the ORF 5 gene of 56 representative PRRSV-positive isolates. Through phylogenetic and molecular analyses, we evaluated the genetic diversity and evolutionary patterns of the virus. Our results offer valuable insights for improving regional strategies to control PRRSV.

## 2 Materials and methods

### 2.1 Sample collection and processing

From 2023 to 2024, we collected 499 clinical samples (lung tissue, lymph nodes, placental tissue, and whole blood) from 101 swine farms across 19 prefecture-level cities in Sichuan Province (Bazhong City, Chengdu City, Dazhou City, Deyang City, Ganzi Prefecture, Guang’an City, Guangyuan City, Leshan City, Liangshan Prefecture, Luzhou City, Meishan City, Mianyang City, Nanchong City, Neijiang City, Suining City, Ya’an City, Yibin City, Zigong City, and Ziyang City). All samples were collected from pigs exhibiting clinical symptoms of PRRSV infection, including elevated body temperature (>40°C) and reduced appetite. The samples were transported on ice and maintained under cold chain conditions until processing (see Supplementary Table S1).

For preparation, 0.5 g of each individual tissue sample was homogenized in sterile PBS (1:5 w/v) in 1.5 mL microcentrifuge tubes, subjected to three freeze–thaw cycles (−80°C), and centrifuged at 12,000×*g* for 10 min at 4°C. The clarified supernatant was either processed immediately or stored at −80°C.

### 2.2 RNA extraction and cDNA synthesis

Total RNA was extracted using the TRIzol reagent (Life Technologies, Carlsbad, USA). The RNA quality was assessed by spectrophotometry (NanoDrop), with A260/A280 ratios ranging from 1.8–2.0 considered acceptable. First-strand cDNA was synthesized from 500 ng of total RNA using random priming (Vazyme, Nanjing, CN), following the manufacturer’s protocol. The cDNA products were stored at −20°C until further use. For comparison, the live attenuated vaccine strain TJM-F92 (Zoetis Biology Co., Ltd., Girona, Spain) and PBS were used as positive and negative controls, respectively.

### 2.3 PRRSV detection and amplification of the complete ORF5 gene

To detect PRRSV in clinical samples, real-time PCR was performed using the detection kit for PRRS produced by Vazyme Biotech Co., Ltd. (Vazyme, Nanjing, China), following the manufacturer’s instructions. Amplification was carried out using the LightCycler 96 Real-Time PCR Detection System (LightCycler^®^ 96 System, Roche, USA).

The primers used to amplify complete ORF5 gene are summarized in Table 1. The amplification reaction system (50 μL in the total system) was constructed as follows: 25 μL of 2 × Phanta Flash Master Mix (Dye Plus) (Vazyme, Nanjing, CN), 2.5 μL of forward and reverse primers, 5 μL of cDNA template, and 15 μL of DEPC water. Amplification was performed under the following reaction conditions: 5 min at 94°C for 1 cycle; 30 cycles of 30 s at 94°C, 30 s at 58°C, and 1 min at 72°C; 10 min of incubation at 72°C for 1 cycle; and a final hold at 4°C. The PCR products were purified using a gel extraction kit (Vazyme, Nanjing, China), then cloned into the pUC-19 vector (Vazyme, Nanjing, China) following the manufacturer’s protocol, and subsequently transformed into DH5α competent cells (Vazyme, Nanjing, China). Three clones of each PCR product were then analyzed via Sanger sequencing by Sangon BioTech Co., Ltd. (Chengdu, CN). Finally, the assembled sequences were generated utilizing SeqMan software (Version 7.0; DNASTAR, USA).

**Table 1:** ORF5 whole gene amplification primers.

Primer	Sequence (5′-3′)	Fragment length (bp)	Annealing temperature (°C)
HP-PRRSV ORF5-F	ATGTTGGGGAAATGCTTGAC	610 bp	55
HP-PRRSV ORF5-R	CTAAGGACGACCCCATTGTTC		
NADC30 Like-PRRSV ORF5-F	ATGTTGGGGAAATGCTTGAC	604 bp	55
NADC30 Like-PRRSV ORF5-R	CTATGGACGACCCCATTGTTC		
EU-PRRSV ORF5-F	ATGAGATGCTCTCACACATCGG	606 bp	56
EU-PRRSV ORF5-R	CTAGGCCTCCCATTGCTCGGC		

### 2.4 Phylogenetic and molecular characterization

For comparative analysis, we included 56 representative PRRSV reference sequences retrieved from the GenBank database. Detailed information is provided in Table 2. Sequence alignment was performed using a two-step approach: (1) Nucleotide (nt) and deduced amino acid (aa) sequences were initially aligned using the Clustal W algorithm implemented in MegAlign (DNASTAR), followed by (2) manual refinement to ensure proper codon alignment. Phylogenetic reconstruction was conducted using Molecular Evolutionary Genetics Analysis (MEGA) software version 7.0, employing the maximum likelihood method with the General Time Reversible model incorporating gamma distribution and invariant sites (GTR + G + I), which was determined as the optimal substitution model through model testing. Branch support was assessed with 1,000 bootstrap replicates. The resulting phylogenetic tree was visualized and annotated using iTOL v6.9.1 (Interactive Tree of Life). For structural characterization, the GP5 protein sequences were analyzed using the ClustalW program in DNAStar v7.0 to identify conserved domains and mutation patterns.

**Table 2:** The reference strain information of PRRSV.

No.	Virus strain	Origin	Accession no.	No.	Virus strain	Origin	Accession no.
1	VR-2332	USA	AY150564	29	TJ	China	EU860248
2	RespPRRs MLV	USA	AF066183	30	HUN4	China	EF635006
3	BJ-4	China	AF331831	31	TJbd14-1	China	KP742986
4	R98	China	DQ355796.1	32	TJnh1501	China	KX510269
5	GS2003	China	EU880442	33	Lelystad virus (LV)	Europe	M96262
6	QYYZ	China	JQ308798	34	BJEU06-1	China	GU047344
7	GM2	China	JN662424	35	NMEU09-1	China	GU047345
8	FJFS	China	KP998476	36	HKEU16	China	EU076704
9	NADC30	USA	JN654459	37	MLV-DV	Netherlands	KJ127878
10	JL580	China	KR706343	38	8-AH11	Czech Republic	KJ614497
11	CHsx1401	China	KP861625	39	NPUST4028	China	OM677758
12	FJ1402	China	KX169191	40	EU-VN05	Viet Nam	OR135693
13	HENAN-XINX	China	KF611905	41	JBNU-PK	South Korea	OQ849109
14	IA/2014/NADC34	USA	MF326985	42	EuroViet-03	Viet Nam	MG251835
15	FJ0908	China	MK202794	43	DK111-92	Denmark	AJ223078
16	RFLP 1–4-4	USA	MW887655	44	Amervac	Spain	GU067771
17	D21-015770	USA	OL963979	45	SCABTC-MY01	China	OR540434
18	D20-041505	USA	OL963972	46	SCABTC-CD05	China	OR540417
19	SCcd2020	China	MW803134	47	SCABTC-MS05	China	OR540433
20	FJGD01	China	OL310959.1	48	SC-2020-1	China	MW115431
21	JS2021NADC34	China	MZ820388.1	49	NEBIH	Hungary	OR143102
22	CH-1a	China	AY032626	50	IV3140	South Korea	DQ355821
23	CH-1R	China	EU807840	51	AN1111	Thailand	KJ954164
24	JXA1	China	EF112445	52	SHE	China	GQ461593
25	JXA1 P80	China	FJ548853	53	NVDC-NM1	China	JX187609
26	JXA1-P170	China	JQ804986	54	ZD-1	China	OP355712
27	JXA1-P120	China	KC422727	55	HUN60077	Hungary	MK167464
28	WUH4	China	JQ326271	56	FJ0603	China	HM114313

## 3 Results

### 3.1 The lineage 1.8 is the dominant epidemic lineage in the Sichuan Province from 2023 to 2024

**Figure 1:**
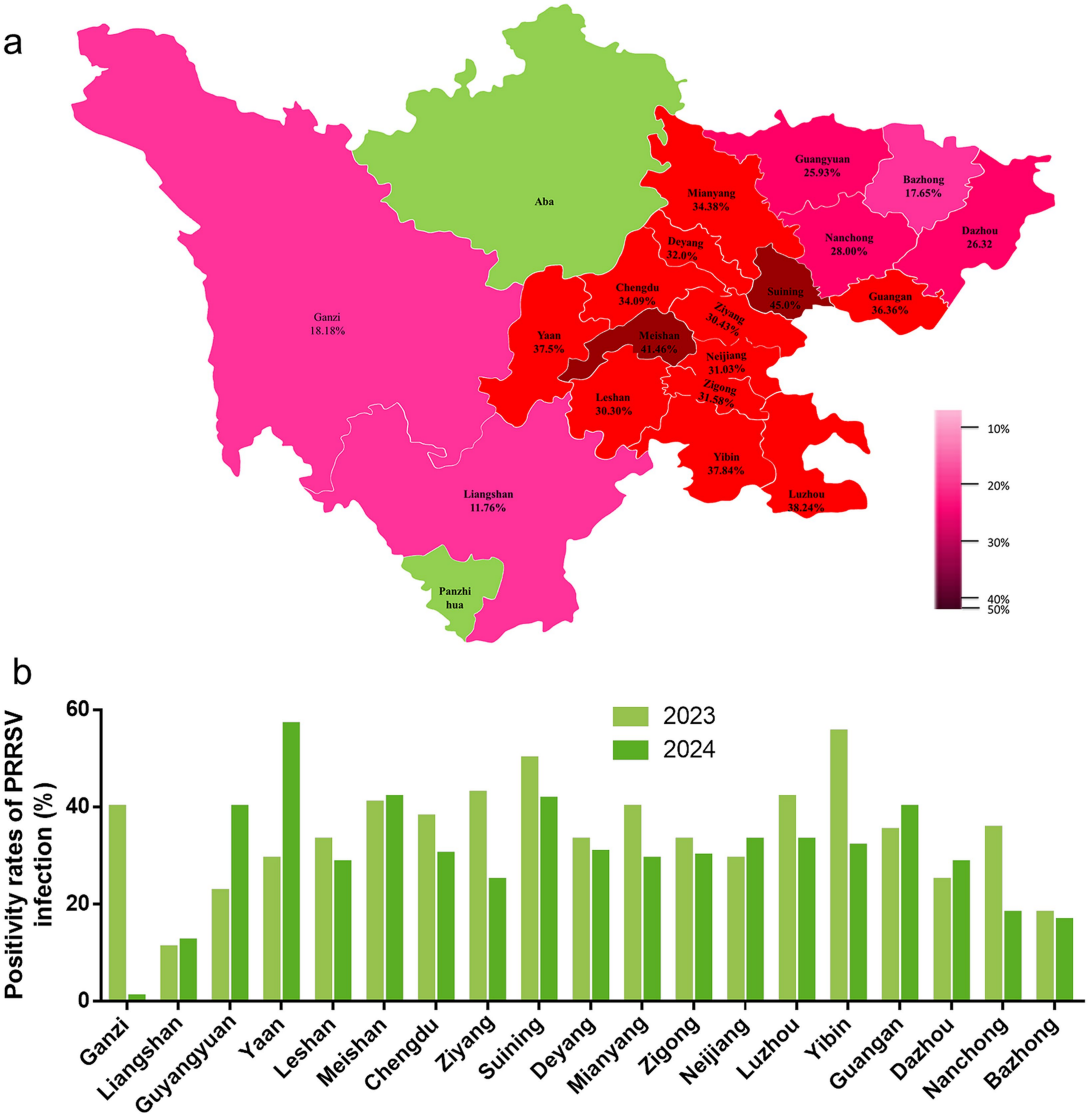
Distribution of PRRSV detection rate in Sichuan Province. **(a)** The distribution of PRRSV detection rates across 19 regions in Sichuan Province from 2023 to 2024. The pink areas represent the regions involved in testing, while the green areas indicate those not participating in the detection, and the numbers indicate the PRRSV prevalence in different cities. **(b)** The prevalence from 2023 to 2024 in 19 regions, where light green represents 2023 and dark green represents 2024.

This study examined 499 clinical samples and detected a total of 162 PRRSV-positive samples with an overall prevalence of 32.46%. The prevalence was 34.11% (88/258) in 2023 and 30.70% (74/241) in 2024, showing a slight downward trend. Regional distribution analysis (Table 3, Figure 1) revealed significant differences in prevalence among 19 prefectures, specifically, Bazhong City (17.65%), Chengdu City (34.09%), Dazhou City (26.32%), Deyang City (32.00%), Ganzi Prefecture (18.18%), Guang’an City (36.36%), Guangyuan City (25.93%), Leshan City (30.30%), Liangshan Prefecture (11.76%), Luzhou City (38.24%), Meishan City (41.46%), Mianyang City (34.38%), Nanchong City (28.00%), Neijiang City (31.03%), Suining City (45.00%), Ya’an City (37.50%), Yibin City (37.84%), Zigong City (31.58%), and Ziyang City (30.43%).

**Table 3:** Information on samples collected in Sichuan province between 2023 and 2024 and results of PRRSV detection.

Cities	2023	2024	Total
Ganzi	2/5(40.0%)	0/6(0.00%)	2/11(18.18%)
Liangshan	1/9(11.11%)	1/8(12.5%)	2/17(11.76%)
Guyangyuan	5/22(22.73%)	2/5(40.0%)	7/27(25.93%)
Yaan	5/17(29.41%)	4/7(57.14%)	9/24(37.50%)
Leshan	4/12(33.33%)	6/21(28.57%)	10/33(30.30%)
Meishan	9/22(40.91%)	8/19(42.11%)	17/41(41.46%)
Chengdu	8/21(38.10%)	7/23(30.43%)	15/44(34.09%)
Ziyang	3/7(42.86%)	4/16(25.0%)	7/23(30.43%)
Suining	4/8(50.00%)	5/12(41.67%)	9/20(45.00%)
Deyang	4/12(33.33%)	4/13(30.77%)	8/25(32.00%)
Mianyang	6/15(40.00%)	5/17(29.41%)	11/32(34.38%)
Zigong	3/9(33.33%)	3/10(30.00%)	6/19(31.58%)
Neijiang	5/17(29.41%)	4/12(33.33%)	9/29(31.03%)
Luzhou	8/19(42.11%)	5/15(33.33%)	13/34(38.24%)
Yibin	5/9(55.56%)	9/28(32.14%)	14/37(37.84%)
Guangan	6/17(35.29%)	2/5(40.00%)	8/22(36.36%)
Dazhou	3/12(25.00%)	2/7(28.57%)	5/19(26.32%)
Nanchong	5/14(35.71%)	2/11(18.18%)	7/25(28.00%)
Bazhong	2/11(18.18%)	1/6(16.67%)	3/17(17.65%)
Total	88/258(34.11% [28.4–40.2%])	74/241(30.71% [25.1–36.8%])	162/499(32.46% [28.4–36.8%])

Values in square brackets indicate 95% confidence intervals calculated by the exact binomial method.

**Figure 2:**
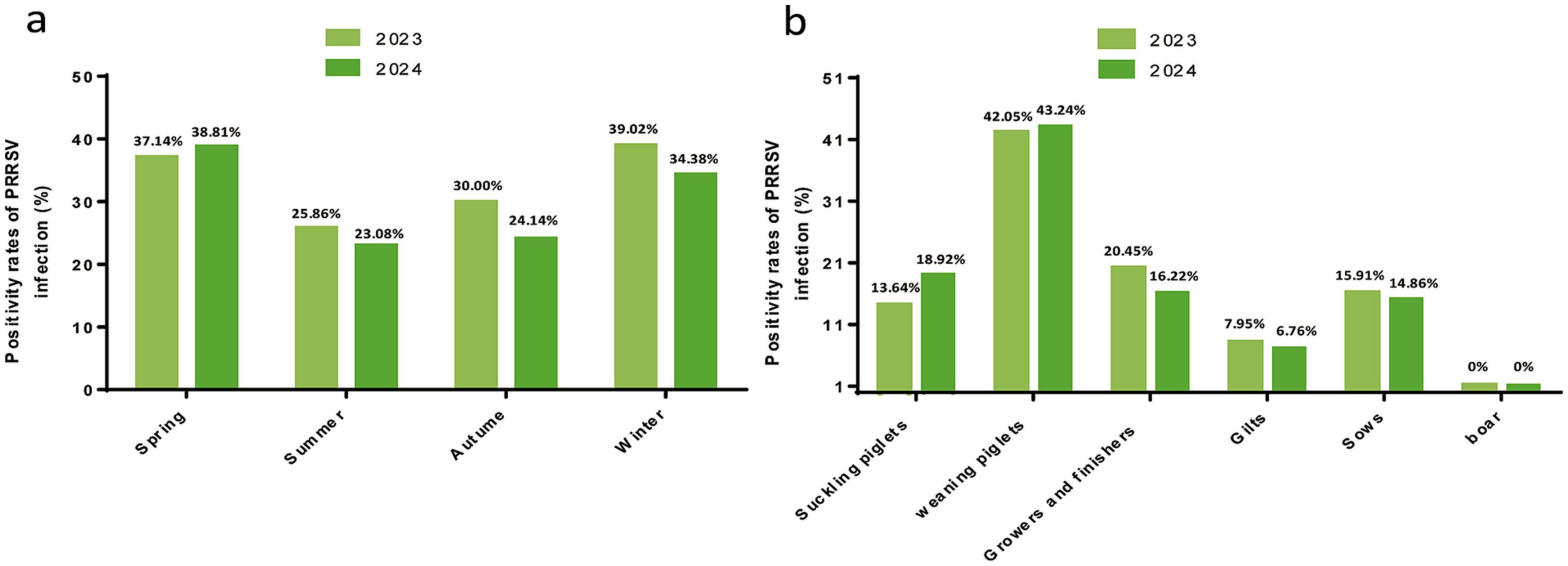
Detection of PRRSV infection in pigs in Sichuan Province. **(a)** PRRSV positivity rates in different seasons. Seasons were defined as winter (December–February), spring (March–May), summer (June–August), and autumn (September–November). The corresponding prevalences were labeled in the histogram. **(b)** PRRSV positivity in pigs at different swine herds.

In terms of seasonal epidemic characteristics, the prevalence in spring 2024 increased slightly compared with that in 2023, but it was lower in summer and autumn. Analysis of prevalence among different pig populations showed that weaning piglets had the highest prevalence (>40%), followed by growers and finishers pigs (16–20%), whereas no positive samples were detected in boar semen. Among the 101 tested pig farms, 55 were positive for PRRSV, with a farm-level prevalence of 54.46% (Figure 2).

The results of the genotype analysis revealed that among the 162 positive samples, the NADC30-like strain accounted for the highest proportion (44.44%, 72/162), followed by the classical PRRSV strain (23.46%, 38/162). The detection rates of HP-PRRSV (11.73%, 19/162), the NADC34-like strain (6.79%, 11/162), and the recombinant QYYZ-like strain (5.56%, 9/162) were relatively low. Notably that PRRSV-1 was detected only sporadically in local areas, with a prevalence of 8.02% (13/162). In particular, both the HP-PRRSV and PRRSV-1 were detected simultaneously in the same sample from a diseased pig farm, suggesting the presence of mixed infection. Epidemiological distribution characteristics revealed the coexistence of multiple strains and mixed infections with different genotypes, with the NADC30-like strain (lineage 1.8) being the predominant epidemic strain, and PRRSV-1 infections exhibiting sporadic characteristics.

### 3.2 Sequencing and phylogenetic analysis

To delve deeper into the genetic variation characteristics of PRRSV from 2023 to 2024, this study selected 1–2 positive samples from each of 55 PRRSV-positive farms for sequencing. As a result, 56 high-quality sequences were obtained for genetic evolution analysis, including 6 sequences of PRRSV-1 strains and 50 sequences of PRRSV-2 strains.

**Figure 3:**
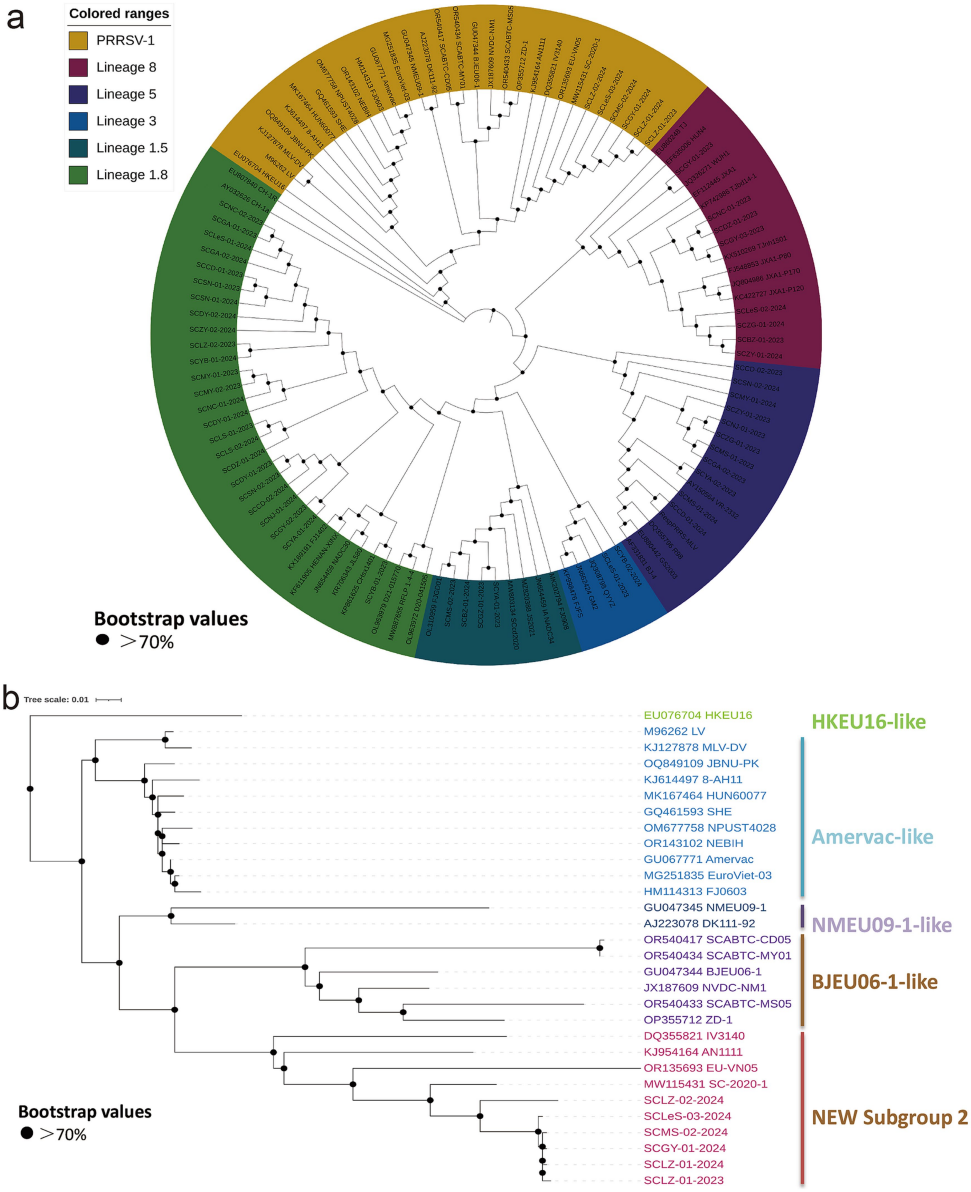
Phylogenetic analysis based on PRRSV ORF5 gene sequences. The phylogenetic tree was constructed using the Neighbor-Joining method in MEGA 7 software. The GTR + G + I model was adopted as the best-fit substitution model, and 1,000 bootstrap replicates were performed. **(a)** Genetic evolution analysis was conducted on 56 sequences obtained in this study and reference sequences, classifying PRRSV into six categories: PRRSV-1 (brown), lineage 1.8 (light green), lineage 1.5 (light blue), lineage 3 (dark blue), lineage 5 (purple), and lineage 8 (pink). **(b)** Evolutionary analysis of six PRRSV-1 sequences obtained in this study and reference strains revealed that the six sequences formed a new lineage.

Analysis of the ORF5 sequences of the 6 PRRSV-1 strains (with a gene length of 606 bp) revealed nt homology of 94.9–99.7% among the strains. The nt homologies with the reference strains BJEU06 and NMEU09 were 85.3–85.5% and 83.8–84.3%, respectively, and 83.8–84.8% with previously isolated strains from Sichuan. Phylogenetic tree analysis indicated that the strains isolated in this study clustered with the early Sichuan isolate SC-2020-1, forming a new subgroup (Figure 3b).

The remaining 50 strains all belonged to PRRSV-2, with nt lengths ranging from 600 to 603 bp. Specifically, 11 strains clustered with the Classical strain, sharing a nt homology of 97.2–100%. Eight sequences grouped with the HP-PRRSV strains, presented nt homology of 98–99.5%. Twenty-five strains clustered with NADC30-like strains, exhibiting nucleotide (nt) homology ranging from 86.4 to 100%. Notably, 15 out of 25 strains harbored a three-nucleotide deletion at positions 94–96 in their sequences. Four strains were grouped with NADC34-like strain, with nt homology ranging from 93.9 to 96.2%. Finally, two strains clustered with the recombinant strain QYYZ-like, demonstrating nt homology of 89.2–92% (Figure 3a).

### 3.3 Deduced amino acid sequence analysis of the GP5 protein

**Figure 4:**
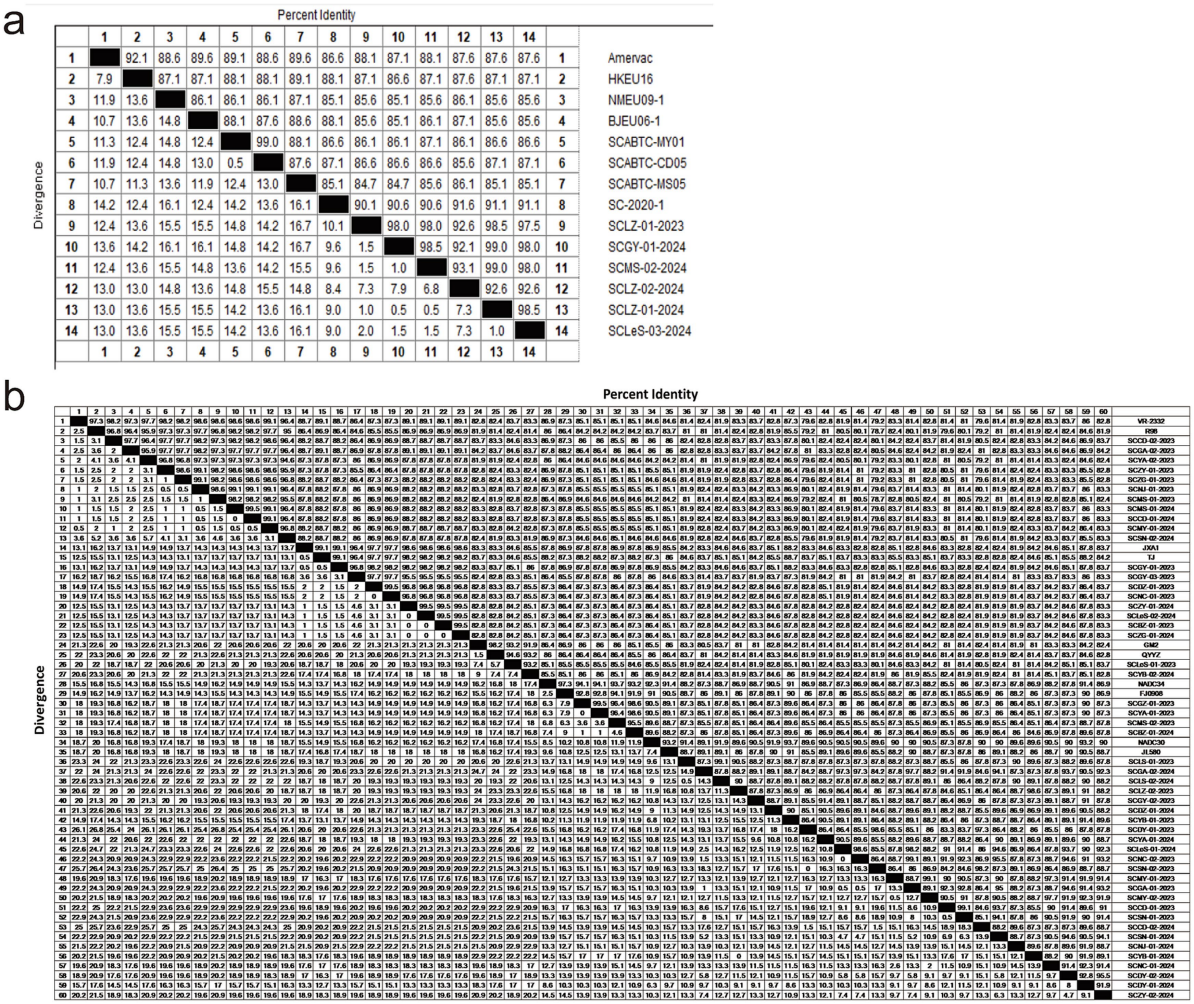
Analysis of PRRSV aa homology differences using the MegAlign program in DNAstar software; **(a)** The aa homology of PRRSV-1 GP5 protein; **(b)** The aa homology of GP5 proteins from different lineages in PRRSV-2.

**Figure 5:**
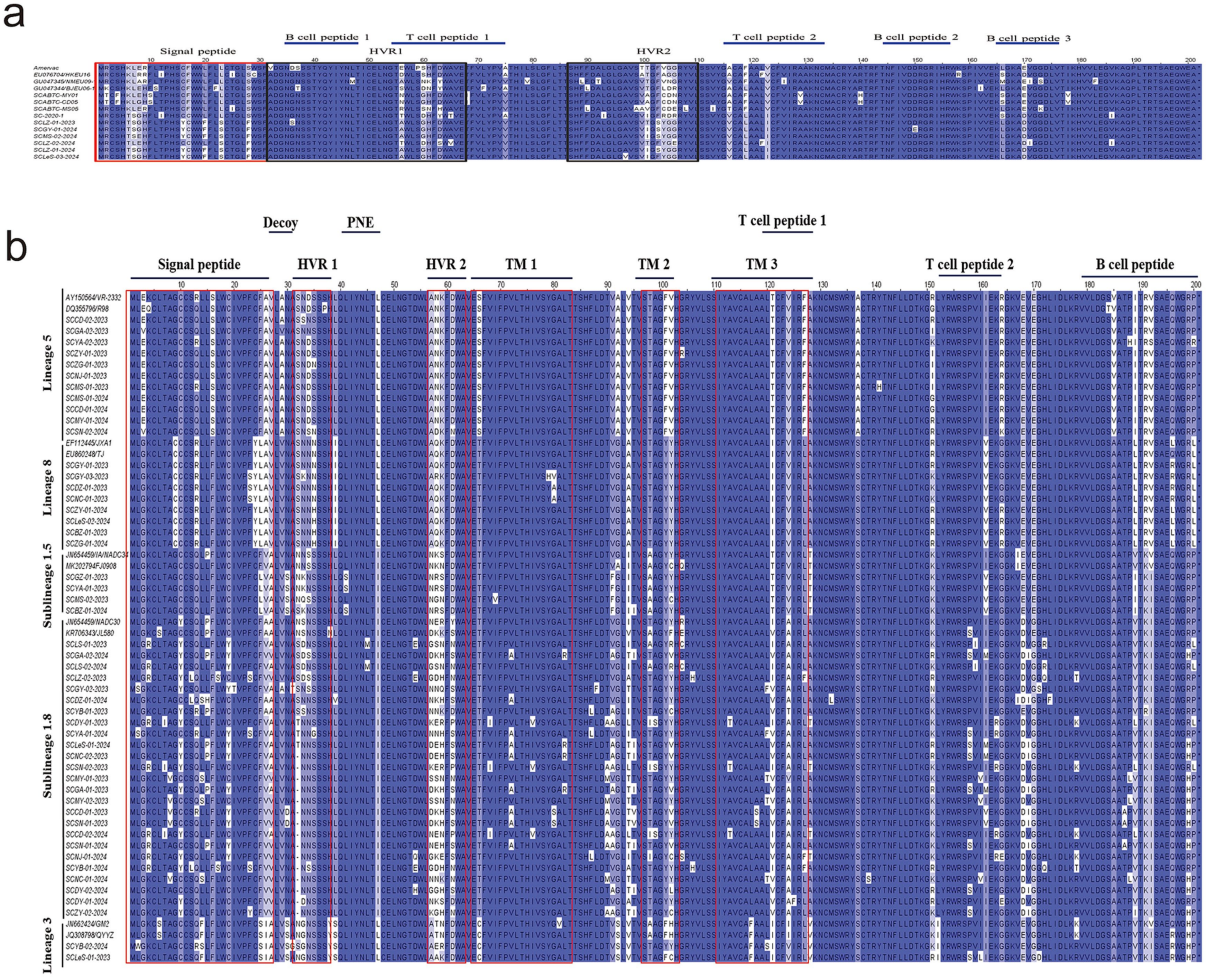
aa analysis of PRRSV GP5 protein (using Jalview-2 for multiple amino acid sequence alignment). **(a)** The aa variability of PRRSV-1 GP5 protein was analyzed, where the red box indicates the signal peptide region, the black box marks the hypervariable regions 1 and 2, and the T-cell and B-cell epitopes are identified with blue lines on the graph; **(b)** The aa variability of PRRSV-1 GP5 protein was conducted. The red boxes identify the signal peptide region, hypervariable regions 1 and 2, and transmembrane regions 1, 2, and 3. The T-cell and B-cell epitopes are labeled above the figure.

First, we analyzed the aa homology of six sequences of PRRSV-1with reference strains. The homology among these six sequences ranged from 92.1 to 99%, whereas the homology with reference strains was between 85.1 and 91.6% (Figure 4a). Among them, SCLZ-02-2024 presented the highest homology with SC-2020-1. Furthermore, by comparing the aa sequences of the six PRRSV-1 sequences with those of representative strains from Sichuan (SCABTC-MY01, SCABTC-CD05, SCABTC-MS05, and SC-2020-1), we identified notable variations clustered in three key regions. Specifically, in the signal peptide region, there are seven characteristic mutation sites: 6 K → 6 T, 7 L → 7S/L, 16C → 16Y, 17F → 17C, 20 L → 20F, 23 L → 23S, and 28S → 28F. Within Hypervariable Region 1 (HVR1), a mutation was observed at position 56 (N → A). In Hypervariable Region 2 (HVR2), mutations occurred at positions 101 (A/V → I/T) and 103–106 (CDE/N → YGE). Additionally, a significant aa mutation was identified at position 170 (E → D) in the B-cell epitope 3 region. Three characteristic aa mutations were detected in T-cell epitope 2: 116 (A/T → V), 119 (F → L), and 123 (V → I) (Figure 5a).

Regarding the diversity of PRRSV-2 strains, we conducted aa mutation analysis for different lineages separately. Within lineage 1.5, the aa homology between the reference strain sequence and the isolated strain ranged from 91 to 93.5% (Figure 4b). Notably, there was an aa mutation at position 25 (F → L) in the signal peptide region, and two aa mutations at positions 189 (V → I) and 192 (V → I) in the B-cell epitope (Figure 5b).

In lineage 1.8, the strains isolated in this study shared 84.1–93% aa homology with those of NADC30 and JL580 (Figure 4b). There were 78 aa mutation sites, with 15 strains showing a deletion of one aa at position 32. Mutations were present at positions 4 (K → R), 15 (P → L), 19 (C → Y), and 26 (A → V) in the signal peptide, as well as at positions 151 (K → R) and 159 (V → I) in T-cell epitope 2. Additionally, there were five aa mutations in the B-cell epitope located at positions 187 (T → A), 188 (P → L), 189 (V → L), 199 (R → H), and 200 (P → L) (Figure 5b).

Within lineage 3, the aa homology between the isolated strains and the reference strains ranged from 92.5 to 94% (Figure 4b). The analysis of aa differences revealed mutations in both strains. Specifically, in T-cell epitope 1, there was a mutation at aa site 124 (I → V). For T-cell epitope 2, the SCLeS-01-2023 strain presented mutations at positions 151 (K → R) and 158 (P → S), whereas the SCYB-02-2024 strain presented three unique mutation sites in the signal peptide region at positions 2 (L → W) and 13–14 (QF → RS) (Figure 5b).

In lineage 5, the strains in this study shared 95–99% aa homology with those of the reference strain, but they exhibited the fewest aa mutation sites. A prominent mutation occurred at position 151 (R/G → I) within T-cell epitope 2. Compared with the reference strains TJ and WUH1 in lineage 8, the eight strains in this study shared the homology of 95.9–99.1% (Figure 4b). Additionally, there were relatively few aa mutation sites. Specifically, within the signal peptide region, only the SCGY-03-2023, SCDZ-01-2023, and SCNC-01-2023 strains exhibited mutations. In addition, the mutation was observed at the 23rd aa position (F → S), a mutation at the T-cell epitope 2 position 151 (R → K), and individual aa mutations at the B-cell epitope 196 (Figure 5b).

Furthermore, we compared five lineages within PRRSV-2 and identified specific mutation sites. Lineage 1 was characterized by a distinct mutation at position 47 (I → L) compared with other genealogies. Lineage 3 contained multiple aa mutations that differ from other genealogies, including mutations at positions 25 (S → F/L), 26 (I → A/V), 39 (S → L/I), 66 (C → T/S), 92 (S → A/G), 117 (F → L), and 128 (V → T/A). In lineage 5, aa mutations at positions 16 (S → F), 66 (S → T), 137 (A → S), and 185 (V → A) differed from those in the other four lineages. Lineage 8 differed primarily from other lineages at aa position 9 (C → G) (Figure 5b).

## 4 Discussion

PRRS is an infectious disease that has the greatest impact on the economic benefits of the pig farming industry in China (4, 23). It has been prevalent for more than 20 years. Despite widespread vaccination efforts, the emergence of new strains due to their extreme variability, high genetic diversity, and genetic recombination has weakened the cross-protection effects of conventional vaccines, leading to the widespread and difficult-to-control prevalence of PRRSV in China. Long-term epidemiological investigations enable the dynamic monitoring and tracking of genetic variations in PRRSV, facilitating the development of targeted prevention strategies to control the spread and transmission of the virus. This study analyzed the prevalence and strain variation characteristics of PRRSV in pig populations based on the ORF5 gene from 19 regions of Sichuan Province between 2023 and 2024. The results provide insights into the latest genetic variations in PRRSV and inform more reasonable measures to control PRRSV.

In this study, among the 499 clinical samples collected, 162 were positive for PRRSV, with a prevalence of 32.46% (162/499). This rate is lower than the previously reported PRRSV prevalence in Southwest China from 2012 to 2020 and from 2021 to 2023 (52.32%, 282/539; 39.74%, 643/1618) (24, 25), indicating a downward trend in the prevalence of PRRSV in this region in recent years. Notably, PRRSV also has a relatively low prevalence in other regions of China. For example, it is reported that the PRRSV prevalence on farms in Guangxi was 19.57% (3); in Henan and Shanxi, the prevalence was only 16.99% (26). A study conducted in Central China reported a prevalence of 23.98% (27); the prevalence in Northern China was only 25.26% (28), while the prevalence in the Northeast was ranged from 17.54 to 53.33% (29). These data indicate that the prevalence of PRRSV differs across regions and that prevalence vigilance and strengthened monitoring are needed to control its transmission.

The analysis of seasonal patterns revealed that PRRSV is detectable year-round, with winter showing the highest prevalence at 39.02% in 2023, followed by spring at 38.81% in 2024. This epidemiological trend aligns with previous research findings (30), although it differs from a study that identified spring as the season with the highest prevalence (31). PRRSV outbreaks, while not strictly seasonal, often correlate with specific seasons. Colder months, particularly winter and early spring, are associated with increased viral activity and prevalence. This seasonal variation could be influenced by environmental conditions, farming practices, and viral stability across temperatures. Colder weather enhances the environmental stability of the virus and may increase pig stress levels, potentially increasing their susceptibility to infection. Low temperatures also favor virus transmission (32).

When considering different pig stages, weaning piglets exhibited the highest prevalence at 42.59% (69/162), with no positive cases detected among boars and a prevalence of 7.41% (12/162) among gilts. The elevated prevalence in nursery pigs can be attributed to two primary factors: increased infection risk due to waning maternally derived antibodies and immature cellular immunity, peaking 3–6 weeks postweaning, and persistent PRRSV infection resulting from high-density housing and poor ventilation in nursery facilities. Conversely, the low prevalence among replacement sows is attributed to the acclimatization of incoming PRRSV strains and vaccination, which reduces transmission risk within the herd. These findings underscore the increased susceptibility of piglets and nursery pigs, highlighting the need for enhanced protective measures during these critical stages. Recent studies have indicated that the “All In/All Out” (AIAO) weaning strategy can effectively reduce the circulation of PRRSV during the nursery phase, possibly by decreasing cross-infection among pigs of different ages. Delayed weaning or mixing of piglets (such as keeping frail piglets and mixing them with newly weaned piglets) significantly increases the likelihood of virus transmission (33). It is worth noting that there are variations in prevalence rates across different regions, with lower rates observed in mountainous areas primarily due to the effect of geographical isolation.

The phylogenetic tree constructed on the basis of PRRSV-1 and PRRSV-2 sequences (Figure 3) demonstrated that 50 PRRSV-2 strains from Sichuan Province can be classified into five lineages (lineages 1.5, 1.8, 3, 5, and 8). Among these lineages, lineage 1.8 (NADC30-like strain) represents the dominant circulating lineage (accounting for 42.86%), which aligns with the recent PRRSV epidemic trend in China. The characteristic 131-aa deletion in the nsp2 protein of this gene may enhance its replicative fitness (21). The co-circulation of multiple genes is consistent with epidemiological reports from other regions of China and globally (34, 35). Furthermore, within the same region, the prevalent virus strains are gradually changing. From 2012 to 2020, lineage 8 were dominant in Sichuan (24), whereas a notable transition to lineage 1.8 occurred between 2021 and 2023, indicating a significant alteration in circulating genealogies (25). Recent epidemiological surveys of PRRSV revealed that the prevalent lineage in major pig-farming regions of China have gradually shifted from lineage 8 to lineage 1.8. In a few areas, such as Henan and Shanxi Provinces, the lineages 1.8 and 1.5 respectively, predominantly circulate (26), with multiple lineages co-circulating. In Vietnam, however, the prevalent strain of PRRSV remains primarily lineage 8 (36). These variations may reflect differences in vaccination strategies or swine production systems, warranting further investigation. Unexpectedly, we detected concurrent lineage 8 and PRRSV-1 in one farm, potentially linked to cross-regional swine introduction. Furthermore, studies have reported a higher detection rate of PRRSV-1 in China’s original swine farms than in slaughterhouses, indicating that the source of PRRSV-1 in China may still be linked to breed introduction. These findings suggest two key points: (1) Appropriate measures need to be taken for the prevention and control of PRRSV-1, (2) detection during exotic breed introduction and isolated acclimatization are highly essential. These findings clarify the complex epidemiological landscape of PRRSV, emphasizing the need for vaccine development based on local circulating strain characteristics and the formulation of precise regional control strategies. Continuous molecular epidemiological monitoring can establish a dynamic early warning system for circulating strains, providing a scientific basis for vaccine updates and the optimization of prevention and control systems.

The GP5 protein of PRRSV, which serves as the main target of the host immune response, is crucial for vaccine development and investigations into viral pathogenesis mechanisms (37). This protein contains the most prevalent neutralizing epitopes, encompassing a variety of T-cell (119–127 aa and 151–159 aa) and B-cell epitopes (36–52 aa and 168–198 aa) (38, 39). An examination of the amino acid sequences of the GP5 protein across 56 PRRSV strains revealed 78 mutations in the lineage 1.8 (Figure 5b). Notable differences were found in the areas of the signal peptide, B-cell epitopes, and T-cell epitopes among lineages. Specific mutations can act as molecular markers for lineage differentiation; for example, lineage 5 shows distinct amino acid changes at positions 16 (S → F), 66 (S → T), 137 (A → S), and 185 (V → A) compared with the other four lineages. In lineage 8, the main differentiation from other lineages occurs at amino acid position 9 (C → G). Lineage 1 is defined by a unique mutation at position 47 (I → L) in contrast to other genealogies. Lineage 3 displays several amino acid variations that set it apart from other lineages, including changes at positions 25 (S → F/L), 26 (I → A/V), 39 (S → L/I), 66 (C → T/S), 92 (S → A/G), 117 (F → L), and 128 (V → T/A). Furthermore, the emergence or loss of previously identified N-glycosylation sites on the GP5 protein has been noted. Research has suggested that alterations in these glycosylation sites can either increase or reduce a strain’s virulence (40, 41). In our investigation, 15 sequences exhibited aa deletions at position 32 in lineage 1.8. Nevertheless, earlier studies indicated deletions primarily at positions 33 or 34 (42–45), presenting a discrepancy with our results but agreeing with findings by Zhang (46). The role of these deleted aa in immune evasion and the virulence of the strain is still uncertain, and future research utilizing reverse genetics may be pursued. Furthermore, research has shown that mutations at positions R13 and 151R are associated with increased strain virulence (47). In this investigation, five of sequences exhibited mutations at position 151 in lineage 1.8, whereas no mutations were detected at position 13. Potential effect of these mutation sites on virulence needs further investigation.

In conclusion, we acquired both the prevalence rates and the molecular characteristics of PRRSV across 19 areas within Sichuan Province. At present, the lineage 1.8 continues to be the most common lineage, yet the increasing prevalence of PRRSV-1 required further investigation and ongoing monitoring. Additionally, distinct mutations and deletions in the 32 aa of PRRSV GP5 have been noted various strains, contributing to the genetic variation of PRRSV. These findings provide crucial information and insights that improve our understanding of epidemiology and could assist in understanding the prevalence, genetic features, and the development of detection kits and vaccines, and ultimately help establish effective prevention and control strategies for PRRSV.

## Data Availability

The datasets generated in this study are available and the sequences have been deposited in the GenBank database under the Accession numbers PV832371-PV832426.
